# Cutaneous metastasis of primary urothelial ureteral cancer: an exceptional case with atypical presentation^؆^

**DOI:** 10.1016/j.abd.2025.501265

**Published:** 2026-01-05

**Authors:** Martín Céspede-Núñez, Jonathan Stevens, Andrea Solari, Cecilia Jeraldo

**Affiliations:** a Department of Dermatology, Faculty of Medicine, University of Chile, Santiago, Chile; b Service of Dermatology, Hospital del Salvador, Santiago, Chile; c Faculty of Medicine, University of Chile, Santiago, Chile; d Service of Pathology, Hospital del Salvador, Santiago, Chile

Dear Editor,

Cutaneous metastases are an infrequent manifestation of internal malignancies, with an estimated incidence of 2.9%.^1^ Skin involvement in urothelial carcinomas is exceptional, occurring in approximately 1% of cases, either concomitantly with or up to one year after the diagnosis of the primary tumor. Its relevance lies in the ominous survival prognosis it implies if confirmed.^1^

We report the case of an 87-year-old man with a history of right testicular seminoma treated with orchiectomy and underlying alcoholic liver cirrhosis, who presented with one year of recurrent hematuria. A CT urogram revealed a dilatation and an emptying defect in the proximal third of the left ureter, which was suggestive of urothelial carcinoma. Due to the COVID-19 pandemic, the patient returned two years later with a painful, progressively enlarging mass in the left pubic region adjacent to the base of the penis, without bleeding or discharge.

He was referred to the Dermatology Department, where physical examination revealed a pink, exophytic tumor with well-defined borders and surface scaling, measuring 2.5 × 2.0 cm (Fig. 1A). Dermoscopic evaluation showed a central keratin mass on a pink, structureless background, accompanied by white clods, erosions, and sparse fine irregular linear vessels (Fig. 1B). Based on these features, a provisional diagnosis of squamous cell carcinoma was made. However, excisional biopsy followed by histopathological examination revealed an undifferentiated carcinoma (Fig. 2), with positive immunohistochemical staining for GATA-3 (clone L50-823; Fig. 3) and negative staining for CK7 (clone SP52), CK20 (clone SP33), vimentin (clone V9), S-100 (clone 4C4.9), and OCT-4 (clone MRQ-10), findings consistent with a urothelial origin. A subsequent radical nephroureterectomy confirmed a high-grade papillary urothelial carcinoma with probable lamina propria invasion, thus confirming the primary tumor.

**Figure 1:**
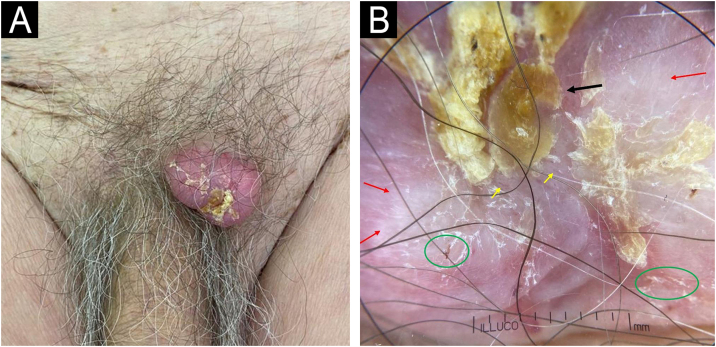
(A) Clinical image: Erythematous tumor with surface scaling located in the pubic region. (B) Dermoscopic image: Central keratin mass (black arrows), whitish areas (red arrows), erosions (green circle), and irregular, fine linear vessels (yellow arrow). (Illuco IDSfe-1100, 10×, polarized mode).

**Figure 2:**
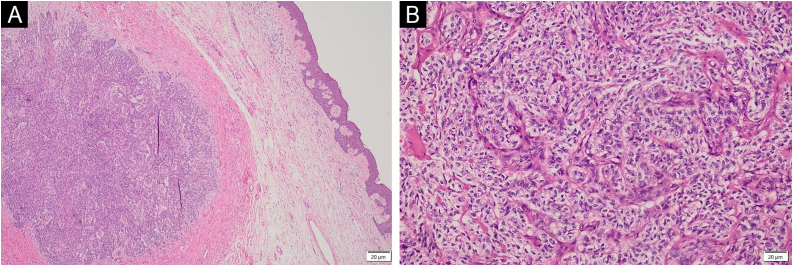
Histopathological study: (A) The epidermis shows hyperkeratosis and parakeratosis. A neoplasm arranged in nests infiltrates the dermis and hypodermis (Hematoxylin & eosin, 4×). (B) The neoplasm is composed of large cells with a high nucleus-to-cytoplasm ratio, moderate pleomorphism, and irregular nuclei. Up to five mitotic figures are observed per 10 high-power fields (Hematoxylin & eosin, 20×).

**Figure 3:**
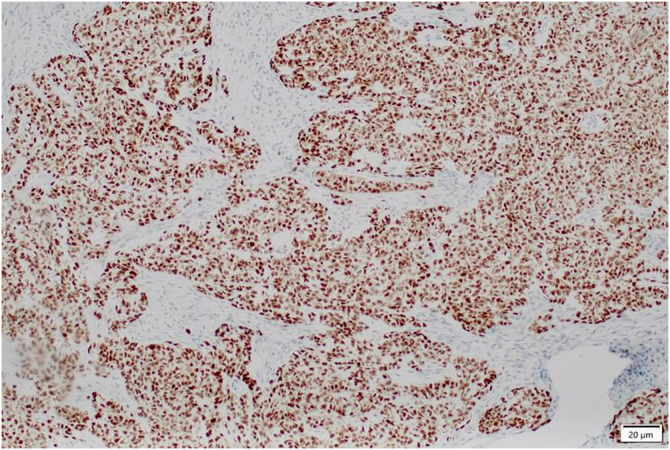
Immunohistochemistry: tumor cells show positive staining for GATA-3.

Bladder cancer is the ninth most common cancer worldwide and the thirteenth leading cause of cancer-related mortality, with an incidence rate of 5.6 per 100,000 individuals.^2^ Approximately 90% of urothelial carcinomas arise from the epithelium lining the urethra, bladder, ureters, and renal pelvis. The 5-year survival rate reaches 96% when diagnosed in early stages, but drops dramatically to 4.6% in the presence of metastases.^3^ These occur in about 5% of cases and typically affect the lymph nodes, bone, liver, and lungs.

Cutaneous metastases from urothelial carcinomas are rare, with bladder cancer accounting for approximately 0.84% of all cutaneous metastases.^4^, ^5^ Dissemination may occur via direct extension, lymphatic or hematogenous spread, or iatrogenic implantation during surgery ‒ the latter being the most frequently reported mechanism.^3^, ^6^ Risk factors include muscle invasion, high histologic grade, poor differentiation, and large tumor size.^7^ Clinically, three patterns of presentation have been described: infiltrative plaques or nodules, sclerotic lesions, and inflammatory-appearing lesions.^8^ The classic manifestation is a multinodular plaque on the anterior abdominal wall.^9^

Diagnosis is confirmed by histological examination with immunohistochemical support, with GATA-3 serving as a reliable marker of urothelial origin. Coexpression of CK7 and CK20 is observed in 89% of cases,^7^ while Uroplakin III positivity has been reported in 50%‒80% of cutaneous metastases from urothelial carcinomas.^1^ First-line treatment is surgical, when possible, and the first-line chemotherapy typically consists of gemcitabine/cisplatin-based chemotherapy, with methotrexate/vinblastine/doxorubicin/cisplatin as a second-line option.^6^, ^10^

In conclusion, reports of cutaneous metastases from urothelial carcinoma are scarce and mostly limited to bladder cancer. To our knowledge, no previous cases of cutaneous metastasis originating from a ureteral urothelial carcinoma have been reported. Clinical presentation is often nonspecific and can mimic primary skin malignancies, inflammatory conditions, or other dermatoses.^9^ Accurate identification of these lesions and other potential metastatic sites is essential, as the prognosis remains poor regardless of the treatment, which is typically palliative.^6^, ^9^

This case represents the first report of a cutaneous metastasis from a primary ureteral urothelial carcinoma. Notably, its clinical presentation mimicked a squamous cell carcinoma, diverging from the classic description of anterior abdominal wall multinodular plaques.

## ORCID ID

Martín Céspede-Núñez: 0009-0003-0634-4306

Jonathan Stevens: 0000-0002-0100-528X

Andrea Solari: 0000-0002-4848-9488

Cecilia Jeraldo: 0000-0001-9462-1235

## Research data availability

Does not apply.

## Financial support

This research did not receive any specific grant from funding agencies in the public, comercial, or not-for-profit sectors.

## Authors' contributions

Martín Céspede Núñez: Data collection; writing of the manuscript and critical review of important intellectual content; critical review of the literature; final approval of the final version of the manuscript.

Jonathan Stevens Gonzalez: Data collection; writing of the manuscript and critical review of important intellectual content; intellectual participation in the therapeutic conduct of the studied case; critical review of the literature; final approval of the final version of the manuscript.

Andrea Solari del Sol: Data collection; writing of the manuscript and critical review of important intellectual content; intellectual participation in the therapeutic conduct of the studied case; critical review of the literature; final approval of the final version of the manuscript.

Cecilia Jeraldo Romero: Data collection; writing of the manuscript and critical review of important intellectual content; intellectual participation in the therapeutic conduct of the studied case; critical review of the literature; final approval of the final version of the manuscript.

## Conflicts of interest

None declared.
